# Large-Scale Whole Genome Sequence Analysis of >22,000 Subjects Provides no Evidence of *FMR1* Premutation Allele Involvement in Autism Spectrum Disorder

**DOI:** 10.3390/genes14081518

**Published:** 2023-07-25

**Authors:** Alex Chubick, Evan Wang, Cora Au, Wayne W. Grody, Roel A. Ophoff

**Affiliations:** 1 Department of Human Genetics, University of California Los Angeles, Los Angeles, CA 90095, USAwgrody@mednet.ucla.edu (W.W.G.); 2 Center for Neurobehavioral Genetics, Jane and Terry Semel Institute for Neuroscience and Human Behavior Los Angeles, University of California Los Angeles, Los Angeles, CA 90024, USA; ewangr@g.ucla.edu; 3 Department of Pathology & Laboratory Medicine, University of California Los Angeles, Los Angeles, CA 90024, USA; cau@g.ucla.edu; 4 Department of Pediatrics, University of California Los Angeles, Los Angeles, CA 90024, USA; 5 Department of Psychiatry, Erasmus University Medical Center, 3015 GD Rotterdam, The Netherlands

**Keywords:** Fragile X Syndrome, autism spectrum disorder, *FMR1*, repeat expansion, ExpansionHunter

## Abstract

Expansion of a CGG repeat in the Fragile X Messenger Ribonucleoprotein 1 (*FMR1)* gene on the X chromosome is the cause of Fragile X Syndrome (FXS). The repeat length of unaffected individuals varies between 5–40 repeats, whereas >200 repeats are observed in cases of FXS. The intermediate range between 55–200 repeats is considered the premutation range and is observed in roughly 1:300 females and 1:900 males in the general population. With the availability of large-scale whole genome sequence (WGS) data and the development of computational tools to detect repeat expansions, we systematically examined the role of *FMR1* premutation alleles in autism spectrum disorder (ASD) susceptibility, assess the prevalence, and consider the allelic stability between parents and offspring. We analyzed the WGS data of 22,053 subjects, including 32 FXS positive controls, 1359 population controls, and 5467 ASD families. We observed no *FMR1* full mutation range repeats among the ASD parent-offspring families but identified 180 family members with premutation range alleles, which represents a higher prevalence compared to the independent WGS control sample and previous reports in the literature. A sex-specific analysis between probands and unaffected siblings did not reveal a significant increase in the burden of premutation alleles in either males or females with ASD. PCR validation, however, suggests an overestimation of the frequency of *FMR1* premutation range alleles through computational analysis of WGS data. Overall, we show the utility of large-scale repeat expansion screening in WGS data and conclude that there is no apparent evidence of *FMR1* premutation alleles contributing to ASD susceptibility.

## 1. Introduction

An expansion of a CGG trinucleotide repeat in the 5′ UTR of the Fragile X Messenger Ribonucleoprotein 1 (*FMR1*) gene on the X chromosome is the cause of Fragile X Syndrome (FXS) [1]. Unaffected individuals in the general population carry *FMR1* repeat alleles ranging from 5 to 40 repeats, while repeat lengths of 200 or more cause FXS [2]. Two intermediate allelic ranges of 41–54 and 55–200 repeat units defined as the gray zone and premutation range, respectively, have also been identified and are associated with increased instability in the gametes of a parent, that may result in further de novo allelic expansions transmitted to the offspring [3,4]. While carriers of *FMR1* premutation alleles do not develop FXS, 40% of male and 16–20% of female premutation carriers suffer from fragile X-associated tremor/ataxia syndrome (FXTAS), and 20% of female carriers are reported to develop fragile X-associated primary ovarian insufficiency (FXPOI) [5]. The reported frequency of *FMR1* premutation range alleles in the general population is estimated to be roughly 1 in 300 for females and 1 in 900 for males [6,7,8,9,10]. There have been clinical and community-based studies of *FMR1* premutation carriers that suggest the *FMR1* premutation may increase the risk of Fragile X-associated neuropsychiatric disorders (FXAND) and conditions (FXPAC), including attention deficit disorder (ADHD) and autism spectrum disorder (ASD) [11,12,13,14,15]. However, these studies have relatively small sample sizes, and much larger, more comprehensive studies are required to determine if the *FMR1* premutation has an effect on disorders such as ASD.

Autism Spectrum Disorder (ASD) is a neurodevelopmental disorder characterized by deficits in social communication and repetitive patterns of behavior [16]. Through twin studies, the heritability of ASD has been estimated to be between 50 and 90%, and, to date, more than 100 disease genes and susceptibility loci have been identified [17,18]. ASD patients may present additional medical complications such as intellectual disability or epilepsy, characteristics that are also core observations in patients with FXS [19]. It is, therefore, no surprise that the *FMR1* full mutation range (>200 repeats) that underlies FXS is also reported to be the most common monogenic cause of ASD [20]. However, diagnostic studies of FXS testing in males with ASD have shown *FMR1* full mutation range repeats may be less common in cases of developmental delay, intellectual disability, and ASD [21]. In this study, we focus on the role of *FMR1* premutation alleles in ASD susceptibility by examining the largest available whole-genome sequence (WGS) datasets of ASD families with a total of 20,576 subjects, collected by the Simons Foundation Autism Research Initiative (SFARI), through the Simons Simplex Collection (SSC) [22] and the Simons Foundation Powering Autism Research (SPARK) initiative [23]. These families (*n* = 5467) consist of an ASD proband with both biological parents, and in many instances also an unaffected sibling. *FMR1* full mutation range repeats were not expected to be observed within the SSC dataset as cases of FXS were excluded but may be present in the SPARK cohort. We used an independent WGS control sample of 1359 independent healthy subjects of the Australian Medical Reference Genome Bank (MRGB) to examine the prevalence of *FMR1* premutation alleles as well as a sample of 118 subjects with known *FMR1* repeat expansion status (including permutation and full mutation range carriers) to validate the computational pipeline.

**Figure 1:**
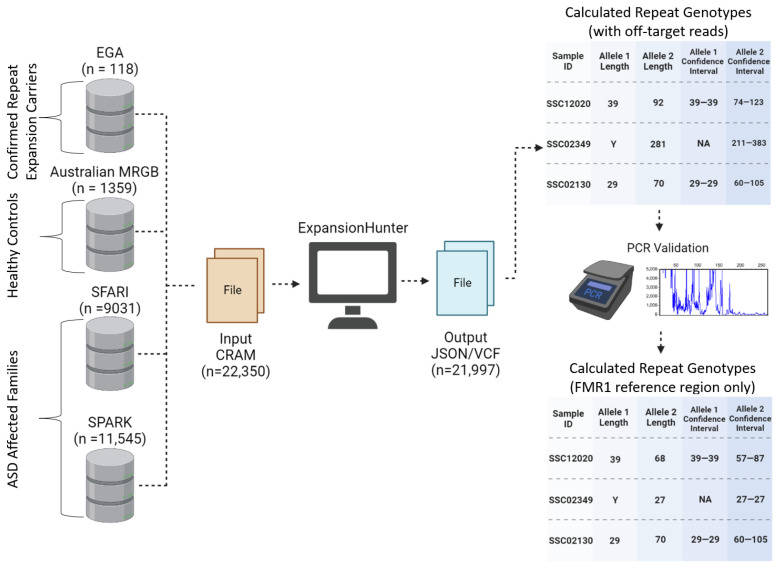
**Workflow of calculating the *FMR1* repeat length of 22,350 samples using ExpansionHunter:** 22,350 sequence alignment files (CRAM format) were downloaded from either the (1) European Genome Archive (EGA), (2) Australian Medical Reference Genome Bank (MRGB), (3) Simons Foundation Autism Research Initiative (SFARI), or (4) the Simons Foundation Powering Autism Research (SPARK) initiative. ExpansionHunter was able to calculate repeat length genotypes for 21,977 samples (98.4%). Four SFARI families with at least one member predicted to carry an expanded repeat were used for molecular validation by PCR. PCR showed overestimation in the repeat lengths calculated by ExpansionHunter, which was shown to be caused by the use of off-target reads in repeat length calculation. To reduce overestimating expanded repeat lengths, samples originally identified to have repeats exceeding the normal range were calculated using only the *FMR1* reference region.

We performed large-scale WGS-based analyses using the computational tool ExpansionHunter [24,25], to examine the *FMR1* CGG trinucleotide repeat in 22,053 subjects (Figure 1). The computational approach correctly identified expanded alleles in the subjects with known expansion status, but the method could not discriminate between premutation or (the much longer) full mutation alleles at this locus. Our analysis of the SSC and SPARK cohorts showed stable transmission of *FMR1* repeat alleles but did not yield evidence for increased burden of *FMR1* premutation (or expanded) alleles in either male or female ASD probands versus unaffected siblings. Molecular validation in ASD families by PCR suggests a degree of overestimation of computationally predicted *FMR1* repeat allele sizes. While ExpansionHunter utilizes repeat sequences in the genome with similar sequences, we restricted our analysis to repeats aligned to the *FMR1* locus which has been reported to reduce overestimation by ExpansionHunter [26]. The transmission patterns we observed of the *FMR1* premutation repeat between parent and offspring also shows that ExpansionHunter does consistently calculate repeat lengths at the *FMR1* locus. With the knowledge that FXS cases were excluded from the SSC cohort and likely biased against in the SPARK cohort, we find no further evidence that *FMR1* premutation alleles play a major role in ASD susceptibility.

## 2. Materials and Methods

### 2.1. Samples with WGS Data

To confirm that our protocol would correctly detect *FMR1* repeat expansions through analysis of whole genome sequence (WGS) data using ExpansionHunter, we obtained PCR-free HiSeqX WGS data on 118 samples with triplet repeat expansions (premutation and full expansions) via the European Genome-phenome Archive (EGA, accession number EGAS00001002462) (Supplementary Table S1). As described, the DNA samples were obtained from Coriell Repository representing subjects with validated repeats in the premutation range (n = 16) and full mutation range (n = 16) and sequenced at an average of 45× coverage [24].

We also obtained access to WGS data of samples from the Simons Simplex Collection that are part of the Simons Foundation Autism Research Initiative (SFARI), the largest ASD collection of trio (proband with both biological parents) and quad (proband with unaffected sibling and both biological parents) families as described before [22]. Briefly, families admitted into the SSC were required to meet metrics related to age such as the proband being between the age of 4 years and 17 years and 11 months when the data were collected and meeting several diagnostic criteria based on Autism Diagnostic Interview-Revised (ADI-R) and Autism Diagnostic Observation Schedule (ADOS) scores. Probands with a known FXS diagnosis, a known genetic risk factor for ASD, were excluded from the repository [22]. Sequence alignment files (CRAM format) from 9031 samples representing a combination of 2380 trio and quad families containing WGS reads aligned to the hg38 reference genome were obtained from SFARI base (https://base.sfari.org accessed on 1 August 2022) (Supplementary Table S1). WGS was performed at 30× coverage on genomic DNA extracted from whole blood as described before [22].

Our analysis also included samples from the Simons Foundation Powering Autism Research (SPARK) initiative, an additional dataset from SFARI, aimed to create the largest recontactable research cohort of families affected with ASD in the United States for longitudinal phenotypic and genomic characterization research studies [23]. Briefly, families admitted into SPARK were required to meet three criteria: (1) have at least one family member with an ASD diagnosis, (2) currently live in the United States, and (3) be able to read and speak English [27]. Samples recruited into the dataset were enriched for affected individuals whose parents were also available to participate. Participants registered for SPARK online (www.SPARKforAutism.org accessed on 1 August 2022) or at a clinical site by completing questionnaires on medical history and social communication, meaning case status in SPARK is based on patient/parent report [27]. Sequence alignment files (CRAM format) from 11,545 samples representing a combination of 3087 trio and quad families containing WGS reads aligned to the hg38 reference genome were also obtained from SFARI base (https://base.sfari.org accessed on 1 August 2022) (Supplementary Table S1). WGS was performed at 30× coverage on genomic DNA extracted from whole blood as described before [28].

For independent reference samples, we utilized the WGS data of 1359 subjects from the Australian Medical Reference Genome Bank (MRGB) available via the European Genome-Phenome Archive (accession code EGAD00001005095) (Supplementary Table S1). Sequence alignment files (CRAM format) were downloaded to determine the frequency of the *FMR1* trinucleotide repeat allele within the MRGB. Data from the MRGB were generated from individuals of European descent, aged 60–95 years, and confirmed to be healthy with no reported history of cancer, cardiovascular disease, or dementia [29]. The MRGB samples were sequenced at an average of >38× coverage. The WGS data were aligned to the hs37d5 reference genome with decoys, with no further processing applied [29].

### 2.2. Genotype Assessment of the CGG Trinucleotide Repeat at FMR1

The length of the CGG trinucleotide repeat in the *FMR1* gene was determined by processing WGS alignment files (formatted in CRAM or BAM format) from the European Genome-Phenome Archive and SFARI base through ExpansionHunter v5.0.0 (https://github.com/Illumina/ExpansionHunter accessed on 1 August 2022) (Supplementary Table S2) [24,25]. Following the established protocol, files processed by ExpansionHunter were run with the reference genome assembly matching the reference of the aligned reads and the variant catalog associated with that reference [24,25]. Additionally, as *FMR1* is located on the X chromosome, ExpansionHunter was run with the “--sex” option as it specifies the sex of the sample and affects the estimated genotype on sex chromosomes. An output JSON and VCF file containing the genotype of *FMR1* were generated for each of the processed files. Additionally, ExpansionHunter estimates a confidence interval for each allele of the genotype using a parametric bootstrap method, and the information is included in the output files (Supplementary Table S2) [24,30].

We also applied Hardy–Weinberg equilibrium testing in females for quality control. Moreover, we used the available pedigree information to examine compatibility with paternal and maternal inheritance patterns. We also utilized a Z-test to determine if there were any differences between the age of parents with offspring who were observed to be de novo for *FMR1* premutation alleles and the age of parents with offspring who inherited premutation range repeat alleles.

### 2.3. PCR Validation of FMR1 Premutation Carriers and Their Pedigrees

CGG repeat lengths in the *FMR1* genes of the subjects were determined by PCR using genomic DNA extracted from whole blood by standard methods [22]. We used the AmplideX *FMR1* PCR reagents (Asuragen, Austin, TX, USA) for amplification and capillary electrophoresis on the ABI 3730 analyzer (ThermoFisher Scientific, Waltham, MA, USA), according to manufacturer specifications. The technique uses repeat-primed PCR (32 cycles) to robustly amplify and accurately size *FMR1* CGG repeats ranging from the normal alleles through premutation alleles to full expansion mutations. We performed the analysis of the capillary electrophoresis profiles using GeneMapper software, Microsatellite subroutine (ThermoFisher Scientific, Waltham, MA, USA), with final results being expressed in base-pair length, trinucleotide repeat number, and normal/premutation/full mutation range based on the accepted cut-offs discussed above.

### 2.4. Repeat Burden Statistical Testing

The burden of repeats in the premutation range of *FMR1* in probands compared to unaffected siblings was determined using Fisher’s exact test. Since *FMR1* is located on the X chromosome and because the prevalence of *FMR1* premutation range alleles has been reported to be different in males and females, we performed sex-specific analyses in which we compared male probands to unaffected male siblings and female probands to unaffected female siblings.

## 3. Results

### 3.1. Validation of FMR1 Repeat Alleles Calculated by ExpansionHunter

**Figure 2:**
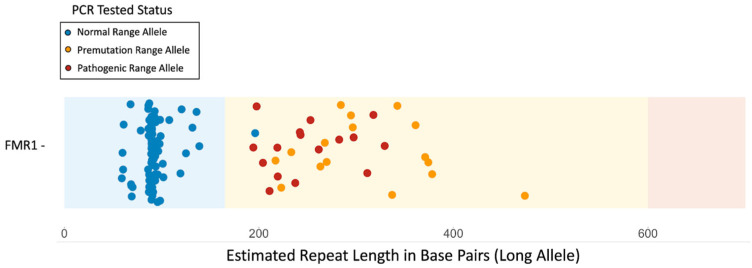
**Validating repeat lengths calculated by ExpansionHunter.** 118 samples identified to have expanded repeats at loci associated with eight different diseases were processed through ExpansionHunter. ExpansionHunter correctly identified all samples with expanded *FMR1* repeat lengths, however, discrepancies were observed in the exact length of the repeats as the samples known to be carriers of full mutation range repeats were classified as being in the premutation range by ExpansionHunter.

ExpansionHunter calculated genotypes for all 118 CRAM files downloaded from the European Genome-phenome archive (EGA). We used the *FMR1* repeat lengths calculated by ExpansionHunter to identify the 32 samples that were listed in the Coriell database to have repeats exceeding the normal range of *FMR1* (Figure 2), of which 16 were in the full mutation repeat length. We observed that ExpansionHunter correctly identifies expanded repeat carriers but underestimates the length of repeats in the full mutation range (Figure 2). In the 84 samples observed in the normal range/gray zone by both PCR and ExpansionHunter, we observed a discrepancy in one sample (1/84) observed with an allele size in the gray zone by PCR but in the premutation range by ExpansionHunter. Overall, we observe that ExpansionHunter (i) correctly identifies all expanded premutation and full mutation range alleles but (ii) underestimates the length of alleles in the full mutation range and (iii) in rare instances, overestimates repeat lengths of shorter alleles.

We selected four families (#11372, #11676, #13390, and #14489) from the SSC cohort with at least one member with a premutation range allele, for PCR validation (Table 1). Among the four families, seven individuals were predicted to be premutation carriers by ExpansionHunter. Based on the repeat lengths determined by PCR, three samples were observed to have repeats in the gray zone, and the remaining samples were observed to have normal range repeats. Our comparison of repeat lengths determined by ExpansionHunter and PCR of SSC samples shows that ExpansionHunter may overestimate repeat lengths at the *FMR1* locus.

Off-target reads were observed to contribute to these overestimated read lengths, and we reprocessed the PCR-tested samples through ExpansionHunter only using the *FMR1* reference region (Supplementary Table S3). The results showed that the newly calculated length of the *FMR1* repeat alleles lengths were much closer to the repeat lengths determined by PCR (Table 1). We also reprocessed the 110 samples from the EGA through ExpansionHunter only using the *FMR1* reference region and observed that the calculated repeat lengths were based on reads only from the X chromosome (Supplementary Table S4). To reduce the likelihood of overestimating the repeat length of samples, we reprocessed samples originally determined to have *FMR1* premutation repeats and calculated their repeat length only using the *FMR1* reference region.

### 3.2. Prevalence of Individuals with FMR1 Repeat Alleles 

**Figure 3:**
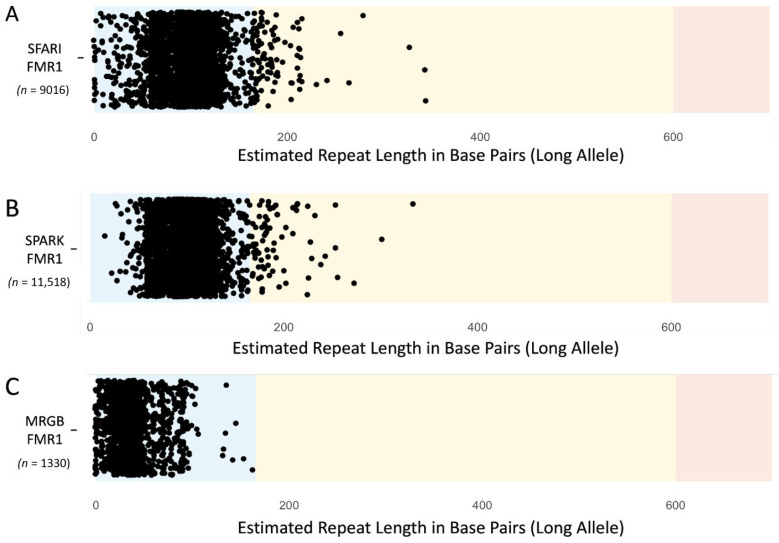
**Estimated repeat length of the *FMR1* trinucleotide repeat in SFARI.** For each subject the longer allele for the *FMR1* genotype is displayed in (**A**) SFARI, (**B**) SPARK, and (**C**) Australia MRGB. Alleles have repeat lengths within the normal (blue), premutation (yellow), or full mutation (red) range. Repeat length estimates were observed to fall within distributions within the normal range (30 bp–120 bp) or around the start of the premutation range.

ExpansionHunter calculated genotypes for 9016 of 9031 (99.8%) of the SSC CRAM files that were downloaded from SFARI. ExpansionHunter does not calculate genotypes for samples with less than 10× coverage or does not possess reads that span the regions in the variant catalog for the *FMR1* locus. We did not observe any samples with repeats that extended into the full mutation range, which is not surprising as families that were identified to have family members with FXS were excluded from the dataset. We also identified 102 samples with repeats in the premutation range of *FMR1* (Figure 3A). Father and male offspring that were carriers of the *FMR1* premutation were observed at a prevalence of 0.93% and 0.71%, while mother and female offspring premutation carriers were observed at a prevalence of 1.78% and 1.28% (Table 2). The prevalence of *FMR1* premutation carriers in the parents of the SSC cohort compared to the prevalence observed in samples from the MRGB (Figure 3C) were significantly higher for mothers (*p*-value = 4.057 × 10^−5^) and nominally significant for fathers (*p*-value = 0.010). We found no evidence that the observed genotype distribution of the *FMR1* premutation in females, diploid for the X chromosome, deviated from what is expected based on the Hardy–Weinberg equilibrium (*p*-value = 0.89, χ^2^ = 0.24). Furthermore, we observed no evidence for bias of *FMR1* premutation allele frequencies in the different sequencing batches of the SSC cohort.

We used ExpansionHunter to calculate an additional 11,520 genotypes from 11,545 (99.8%) CRAM files from the SPARK dataset. We observed no samples with repeats in the full mutation range of *FMR1*. We also identified 78 samples with repeats in the premutation range of *FMR1* (Figure 3B). Father and male offspring that were carriers of the *FMR1* premutation were observed at a prevalence of 0.62% and 0.55%, while mother and female offspring premutation carriers were observed at a prevalence of 0.71% and 0.97% (Table 3). We also observed higher prevalence of *FMR1* premutation carriers in the mothers of the SPARK dataset compared to the prevalence observed in samples from the MRGB (Figure 3C), albeit at nominal significant levels (*p*-value = 0.02) while the prevalence in fathers was observed to be nonsignificant (*p*-value = 0.25). Again, we observed no deviation from what is expected from the Hardy–Weinberg equilibrium (*p*-value = 0.96, χ^2^= 0.08) in female carriers and no evidence for bias of *FMR1* premutation allele frequencies in the different sequencing batches.

### 3.3. Burden of FMR1 Premutation Range Alleles on ASD in Males and Females

In the comparison of male probands and unaffected male siblings in the SSC, we did not observe a significant difference in allele frequencies (*p*-value = 0.48, OR = 0.71). We also observed no significant difference in the allele frequencies (*p*-value = 0.27, OR = 1.73) between female probands and unaffected female siblings in the SSC. In the SPARK dataset, no significant difference was observed between the allele frequencies of male proband and unaffected male siblings (*p*-value = 0.34, OR = 1.81) or female probands and unaffected female siblings (*p*-value = 0.62, OR = 1.30). We also performed a burden test combining the allele frequencies observed in the SSC and SPARK and no significant differences were observed between the allele frequencies of male probands and unaffected male siblings (*p*-value = 0.99, OR = 1.08) or female probands and unaffected female siblings (*p*-value = 0.35, OR = 1.43).

### 3.4. Parental Transmission of FMR1 Premutation Range Alleles

We utilized the *FMR1* repeat length genotypes from the trio and quad families to examine patterns of paternal and maternal inheritance as well as for the identification of putative de novo repeat expansion events. There were 79 families (including 62 quads and 17 trios) with 141 offspring of which 32 (22.7%) inherited the premutation allele from one of their parents (Table 4). Transmissions from mother to male offspring were observed two times more often than transmission from mother to female offspring, which corresponds to the ratio of male and female offspring in the SSC (Table 2). Additionally, we observed 5 offspring (3.5%) with premutation range alleles that are best explained by a de novo expansion of the *FMR1* repeat (Table 4). While we observed that offspring with the premutation more commonly inherit the allele from one of their parents instead of de novo expansions occurring, we also observed 42 offspring (29.8%) who did not inherit a premutation allele from one of their parents who were identified to be a carrier (Table 4). No father-to-male offspring transmission was observed as expected by X-linked inheritance (Table 4).

In the SPARK dataset, we observed within 58 families (including 21 trios and 37 families with four or more members) that 34 offspring out of 99 (34.3%) inherited the premutation allele from one of their parents (Table 5). Similar to the SSC cohort, male probands constitute a majority of the offspring in the SPARK dataset (Table 3). In correspondence, we observed the transmission from mother to male proband to be the most common form of transmission from parent to offspring (Table 5). We also observed three offspring (3.03%) where the expansion of the *FMR1* repeat allele occurred de novo (Table 5). In contrast, we observed 26 offspring with premutation allele sizes (26.3%) who showed no inheritance of premutation range alleles from their parents (Table 5). The patterns of inheritance that we observed in the SPARK dataset were further validated by the fact that no father to male offspring transmissions were observed (Table 5).

In a combined analysis based on the transmission patterns in the SSC and SPARK pedigrees we compared the age of parents who transmitted premutation alleles to their offspring to the age of parents with offspring that possessed premutation alleles best explained by de novo expansion. We observed no significant differences in the age of the parents (*p*-value = 0.98) between the two groups of offspring.

## 4. Discussion

In a large-scale and systematic study of whole genome sequencing (WGS) data of >2200 nuclear families with autism spectrum disorder (ASD) affected probands and unaffected siblings, we found no evidence of increased burden of *FMR1* premutation range alleles in male or female ASD patients compared to unaffected siblings. We applied a conservative approach for association testing by using unaffected siblings instead of unrelated subjects as controls, which is considered more robust against false positive results [31]. Our follow-up results suggest that the computational approach of genotyping *FMR1* repeat alleles may result in some degree of overestimating allele sizes, but we also show in a control sample that true carriers of *FMR1* repeat (premutation) expansions are properly identified. We performed a number of additional quality control steps to confirm that the lack of association between *FMR1* premutation alleles and ASD is the most likely explanation of our study.

The full mutation of the *FMR1* repeat expansion causing FXS is considered to be the most common monogenic cause of ASD accounting for up to 6% of cases [32]. A distinctive exclusion criterion of the Simon Simplex Collection (SSC), however, is the FXS diagnosis of an ASD proband, which has resulted in the absence of full mutation carriers in this cohort. The Simons Foundation Powering Autism Research (SPARK) initiative, an ongoing effort that is also part of the Simons Foundation Autism Research Initiative (SFARI), does not apply the same rule for exclusion, but it is likely that the severe and distinctive phenotype of FXS itself has organically resulted in a total depletion of families with FXS patients. It may therefore not come as a surprise that we did not find evidence for full mutation range *FMR1* alleles in either cohort.

The computational analysis of WGS data resulted in a higher-than-expected number of *FMR1* premutation carriers compared to previous molecular studies with up to six-fold higher prevalence in males (fathers, 0.72%) and four-fold higher prevalence in females (mothers, 1.47%) (Table 2 and Table 3) [33]. The prevalence of female *FMR1* premutation carriers in the SFARI pedigrees was also observed to be significantly higher compared to the Medical Reference Genome Bank (MRGB), which served as an independent control sample of healthy subjects of advanced age. This may suggest that an increased prevalence of premutation alleles is found in ASD families in general, or that the expanded allele is depleted in healthy aged subjects if we assume that technical differences related to WGS data collection are not playing a role in the decreased repeat length observed in the control cohort (Figure 3). We do note that the ExpansionHunter analysis of the gnomAD samples shows a similar prevalence of *FMR1* premutation carriers as we observed in the Simons Simplex Collection (males = 0.70%, females = 0.98%) [26]. We used PCR to validate the *FMR1* premutation repeats identified in a small subset of samples (i.e., four of the 79 quad families with computational evidence of a premutation) from the SSC dataset. Molecular PCR testing showed that none of the expected samples were positive for a premutation range allele but that three of the expected seven samples with expanded alleles carried an allele in the gray zone (Table 1). The lack of PCR validation of permutation alleles suggests the presence of false positives among our computational results which requires further large-scale molecular assessment and advancements of bioinformatic approaches of *FMR1* repeat genotyping.

We consider several reasons for the lack of replicable findings by PCR. First, ExpansionHunter may have overestimated the allele lengths as has been reported for some other repeat loci [26]. We also observed that the confidence intervals estimated by ExpansionHunter broaden with increased repeat unit length (Supplementary Figure S1), which may explain the overestimated repeat lengths calculated in the SFARI cohorts. Moreover, mosaicism, higher sequencing error rates, and GC biases are factors that can contribute to this discrepancy at the *FMR1* locus [24,25]. Studies have also shown that the use of off-target regions in the variant catalog used in the ExpansionHunter algorithm can lead to the overestimation of some genotypes [26]. We observed that off-target reads from chromosomes 2, 6, and 7 lead to an overestimation of the genotypes calculated by ExpansionHunter in the PCR-tested samples (Supplementary Table S3), which suggests similar repeat sequences exist in these chromosomes and should be further explored to determine if these have an effect on ASD or other traits. On the other hand, the stability and consistency we observed in the length of alleles transmitted from parent to offspring in the Simons Simplex Collection suggest that, generally speaking, ExpansionHunter does accurately calculate the repeat length from WGS data. For example, we did not observe instances of father-to-son transmission of expanded alleles, an incompatibility for X-linked alleles, which suggests consistent calling of *FMR1* variants. We also note that the observed *FMR1* premutation genotype distribution in females (with diploid genotypes) did not violate the expected distribution according to the Hardy–Weinberg equilibrium, which suggests that there is no inherent technical deviation in the detection of premutation range alleles in females using ExpansionHunter.

Assuming that most of the observed *FMR1* premutation alleles are correct, we also examined the distribution of segregation and de novo events in the offspring (ASD probands and siblings). Utilizing the family structure, we observed that the vast majority of offspring with premutation alleles inherited the alleles from one of their parents compared to likely de novo expansion events (Table 4 and Table 5). Additionally, we observed that the age of the parents had no effect on whether a premutation allele was transmitted from the parent or occurred de novo in an offspring. Among the transmissions, the mother-to-male-proband transmissions were the most common type of transmission, which is representative of the preponderance of male probands in these ASD cohorts and is expected with a chromosome X-linked locus such as *FMR1*.

There remains some ambiguity of true and false positive cases of *FMR1* premutation carriers identified in our analyses which demonstrates the need for further PCR and computational validation in additional independent datasets. On the other hand, we show that large-scale repeat expansion screening in WGS datasets is possible and that the *FMR1* findings, by and large, follow Mendelian patterns as expected. Given the overall evidence, we conclude therefore that the lack of any evidence for enrichment of *FMR1* repeat premutation allele expansions in ASD probands compared to unaffected siblings in this large study encompassing >22,000 subjects, suggests that *FMR1* premutation alleles do not play a major role in ASD disease risk.

## Figures and Tables

**Table 1:** **Validation of *FMR1* Repeat Alleles Calculated by ExpansionHunter.** The *FMR1* repeat allele estimated by ExpansionHunter was validated by PCR. Experimental validation of these subjects detected no alleles in the premutation range of *FMR1*. Only three of the premutation carriers identified by ExpansionHunter were observed to have longer alleles in the gray zone based on PCR.

Sample ID	Family ID	Family Member	PCR Determined Length Genotype	ExpansionHunter Determined Repeat Length Genotype (with Off-Target Reads)	ExpansionHunter Determined Repeat Length Genotype (FMR1 Reference Region)
SSC02135	11372	Father	Y/23	Y/23	Y/23
SSC02130	11372	Mother	29/48	29/70	29/70
SSC02128	11372	Male Proband	Y/48	Y/46	Y/46
SSC02136	11372	Male Sibling	Y/48	Y/57	Y/51
SSC02349	11676	Father	Y/27	Y/281	Y/27
SSC02338	11676	Mother	29/30	29/30	29/30
SSC02330	11676	Female Proband	27/30	27/30	27/30
SSC02350	11676	Female Sibling	27/29	27/29	27/29
SSC07297	13390	Father	Y/30	Y/30	Y/30
SSC07289	13390	Mother	30/33	30/33	30/33
SSC07282	13390	Male Proband	Y/33	Y/157	Y/71
SSC07298	13390	Female Sibling	30/30	30/30	30/30
SSC12025	14489	Father	Y/30	Y/30	Y/30
SSC12020	14489	Mother	39/46	39/92	38/68
SSC12016	14489	Male Proband	Y/39	Y/128	Y/71
SSC12026	14489	Male Sibling	Y/39	Y/168	Y/72

**Table 2:** **Simons Simplex Collection Samples Processed and Genotyped for *FMR1* CGG Trinucleotide Repeat by ExpansionHunter.** We genotyped the CGG trinucleotide repeat of the *FMR1* locus in 9031 samples from 2380 families using ExpansionHunter. Of the samples that were genotyped, we identified 139 individuals with an *FMR1* premutation allele. In comparison to previous reports, we observe an increased baseline prevalence of premutation carriers in SFARI.

Family Member	Processed Samples	Samples with FMR1 Genotype	FMR1 Premutation Carriers	FMR1 Premutation Non-Carriers	Percentage of FMR1 Premutation Carriers
Father	2364	2362	22	2340	0.93%
Mother	2369	2365	42	2323	1.78%
Male Proband	2060	2055	13	2042	0.63%
Male Sibling	906	905	8	897	0.88%
Female Proband	321	321	6	315	1.87%
Female Sibling	1011	1008	11	997	1.09%
Total	9031	9016	102	8914	-

**Table 3:** **Simons Foundation Powering Autism Research (SPARK) Initiative Samples** Processed and Genotyped for *FMR1* CGG Trinucleotide Repeat by ExpansionHunter. We genotyped the CGG trinucleotide repeat of the *FMR1* locus in 11,545 samples from 3087 families using ExpansionHunter. Of the samples that were genotyped, we identified 92 individuals with an *FMR1* premutation allele. In comparison to previous reports, we observe an increased baseline prevalence of premutation carriers in the SPARK.

Family Member	Processed Samples	Samples with FMR1 Genotype	FMR1 Premutation Carriers	FMR1 Premutation Non-Carriers	Percentage of FMR1 Premutation Carriers
Father	3075	3067	19	3048	0.62%
Mother	3078	3077	22	3055	0.71%
Male Proband	2509	2497	16	2481	0.64%
Male Sibling	1133	1127	4	1123	0.35%
Female Proband	614	614	7	607	1.14%
Female Sibling	1136	1136	10	1126	0.88%
Total	11545	11518	78	11446	-

**Table 4:** **Parental Transmission of the *FMR1* Premutation Allele in SPARK.** Analysis of the calculated repeat length of the *FMR1* trinucleotide repeat in the families within SPARK revealed 35 cases where an offspring was observed to inherit the *FMR1* premutation from one of the parents. The transmission from mother to male proband was most frequently observed, and no father-to-male offspring transmission was observed as expected for X-linked inheritance.

Parent to Offspring Transmission/De Novo Expansion in Offspring	Allele Transmitted	Count	Percentage of Offspring in FMR1 Premutation Families
Mother to Male Proband	Premutation Range Allele	10	7.09%
Mother to Male Sibling	Premutation Range Allele	7	4.96%
Mother to Female Proband	Premutation Range Allele	3	2.12%
Mother to Female Sibling	Premutation Range Allele	6	4.26%
Father to Male Proband	Premutation Range Allele	0	0.00%
Father to Male Sibling	Premutation Range Allele	0	0.00%
Father to Female Proband	Premutation Range Allele	2	1.42%
Father to Female Sibling	Premutation Range Allele	4	2.83%
Mother to Male Proband	Normal Range Allele	19	13.48%
Mother to Male Sibling	Normal Range Allele	14	9.92%
Mother to Female Proband	Normal Range Allele	2	1.42%
Mother to Female Sibling	Normal Range Allele	7	4.96%
Father to Male Proband	Normal Range Allele	0	0.00%
Father to Male Sibling	Normal Range Allele	0	0.00%
Father to Female Proband	Normal Range Allele	0	0.00%
Father to Female Sibling	Normal Range Allele	0	0.00%
Male Proband	de novo	3	2.12%
Male Sibling	de novo	1	0.71%
Female Proband	de novo	0	0.00%
Female Sibling	de novo	1	0.71%

**Table 5:** **Parental Transmission of the *FMR1* Premutation Allele in SFARI.** Analysis of the calculated repeat length of the *FMR1* trinucleotide repeat in the families within SFARI revealed 40 cases where an offspring was observed to inherit the *FMR1* premutation from one of the parents. The transmission from mother to male proband was most frequently observed, and no father-to-male offspring transmission was observed as expected for X-linked inheritance.

Parent to Offspring Transmission/De Novo Expansion in Offspring	Allele Transmitted	Count	Percentage of Offspring in FMR1 Premutation Families
Mother to Male Proband	Premutation Range Allele	15	15.15%
Mother to Male Sibling	Premutation Range Allele	4	4.04%
Mother to Female Proband	Premutation Range Allele	3	3.03%
Mother to Female Sibling	Premutation Range Allele	4	4.04%
Father to Male Proband	Premutation Range Allele	0	0.00%
Father to Male Sibling	Premutation Range Allele	0	0.00%
Father to Female Proband	Premutation Range Allele	3	3.03%
Father to Female Sibling	Premutation Range Allele	5	5.05%
Mother to Male Proband	Normal Range Allele	11	11.11%
Mother to Male Sibling	Normal Range Allele	6	6.06%
Mother to Female Proband	Normal Range Allele	2	2.02%
Mother to Female Sibling	Normal Range Allele	7	7.07%
Father to Male Proband	Normal Range Allele	0	0.00%
Father to Male Sibling	Normal Range Allele	0	0.00%
Father to Female Proband	Normal Range Allele	0	0.00%
Father to Female Sibling	Normal Range Allele	0	0.00%
Male Proband	de novo	1	0.01%
Male Sibling	de novo	0	0.00%
Female Proband	de novo	1	0.01%
Female Sibling	de novo	1	0.01%

## Data Availability

All sequence data used in this study were obtained from publicly available resources as described in the Section 2.

## Supplementary Materials

The following supporting information can be downloaded at: https://www.mdpi.com/article/10.3390/genes14081518/s1, Figure S1: Estimated Confidence Intervals for the FMR1 Repeat; Table S1: CRAM files processed from the Simons Foundation Autism Research Initiative (SFARI); Table S2: Repeat Length Genotypes Calculated by ExpansionHunter for the Simons Foundation Autism Research Initiative (SFARI); Table S3: FMR1 Repeat Length Genotypes Calculated by ExpansionHunter with Off-Target Regions Removed; Table S4: FMR1 Repeat Length Genotypes Calculated for European Genome Archive (EGA) Samples with Off-Target Regions Removed.
