# Supplementary material for: Large-Scale Whole Genome Sequence Analysis of >22,000 Subjects Provides no Evidence of FMR1 Premutation Allele Involvement in Autism Spectrum Disorder
Source: Genes (Basel). 2023 Jul 25;14(8):1518. doi: 10.3390/genes14081518 (PMC10454383; doi:10.3390/genes14081518)
Supplement: Supplementary file 1 [file genes-14-01518-s001.zip › Supplementary_Figure1.pdf]

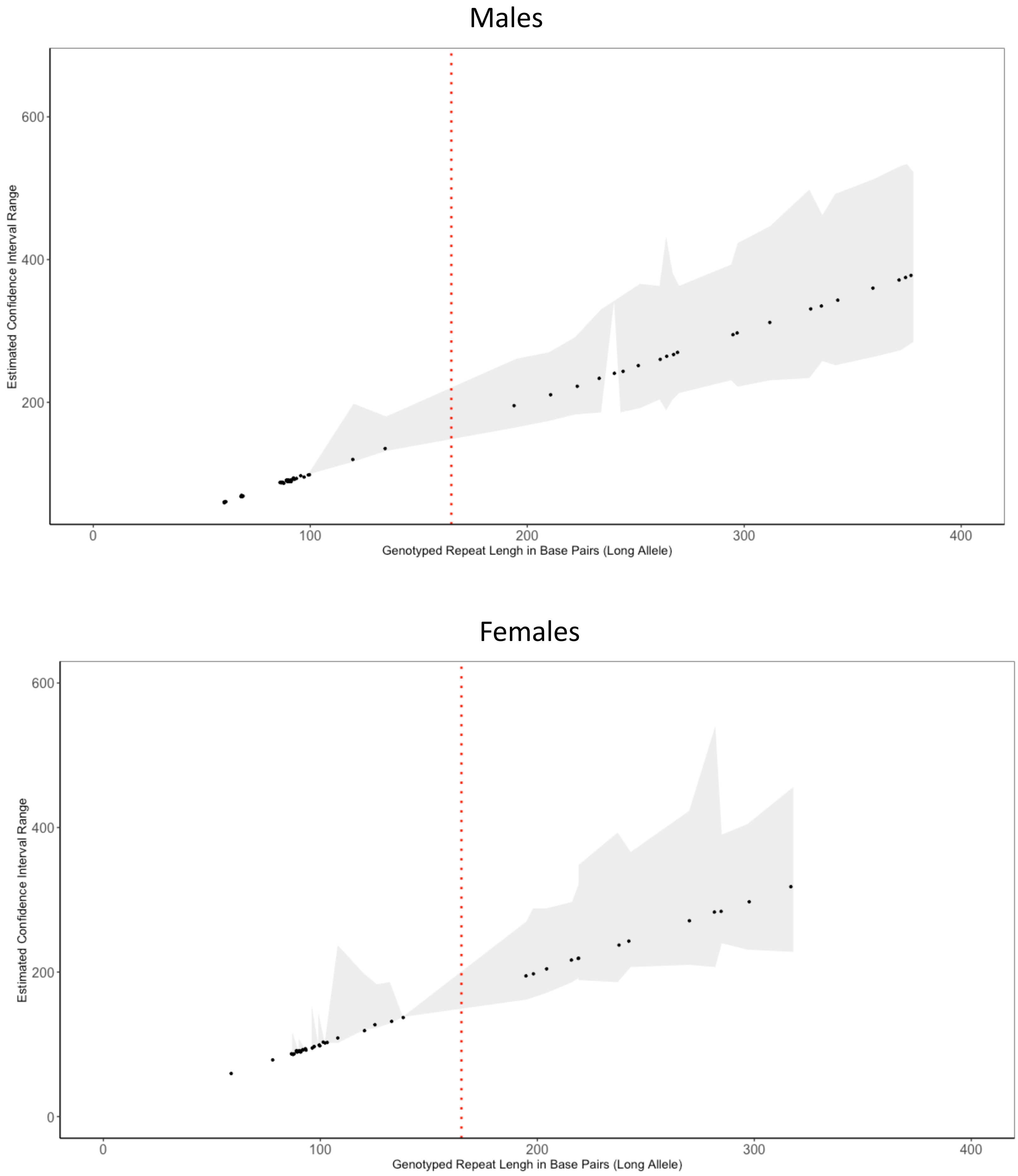

Supplementary Figure 1: Estimated confidence intervals for the repeat length of the long allele for the FMR1 genotype in samples from the European Genome-phenome Archive (EGA). The presence of expanded repeats have been confirmed in the EGA samples and it can be observed that the confidence intervals are narrower for shorter non-pathogenic repeat lengths but broaden with increased repeat units.
